# A New Low Complexity Angle of Arrival Algorithm for 1D and 2D Direction Estimation in MIMO Smart Antenna Systems

**DOI:** 10.3390/s17112631

**Published:** 2017-11-15

**Authors:** Mohammed A. G. Al-Sadoon, Nazar T. Ali, Yousf Dama, Abdulkareim Zuid, Stephen M. R. Jones, Raed A. Abd-Alhameed, James M. Noras

**Affiliations:** 1 School of Engineering and Informatics, University of Bradford, Bradford BD7 1DP, UK; M.A.G.Al-Sadoon@bradford.ac.uk (M.A.G.A.-S.); s.m.r.jones@bradford.ac.uk (S.M.R.J.); J.M.Noras@bradford.ac.uk (J.M.N.); 2 Department of Communication and Informatics Engineering, Basra University College of Science and Technology, Basra 61004, Iraq; kareimzweid@gmail.com; 3 Department of Electrical and Computer Engineering, Khalifa University, Abu Dhabi 127788, UAE; ntali@kustar.ac.ae; 4 Department of Electrical Engineering, Najah National University, Omar Ibn Al-Khattab St., 44859 Nablus, Palestine; yasdama@najah.edu

**Keywords:** direction estimation, smart antennas, wireless communication systems, direct data acquisition (DDA), multiple input multiple output (MIMO), angles of arrival (AOAs), covariance matrix, computational complexity

## Abstract

This paper proposes a new low complexity angle of arrival (AOA) method for signal direction estimation in multi-element smart wireless communication systems. The new method estimates the AOAs of the received signals directly from the received signals with significantly reduced complexity since it does not need to construct the correlation matrix, invert the matrix or apply eigen-decomposition, which are computationally expensive. A mathematical model of the proposed method is illustrated and then verified using extensive computer simulations. Both linear and circular sensors arrays are studied using various numerical examples. The method is systematically compared with other common and recently introduced AOA methods over a wide range of scenarios. The simulated results show that the new method has several advantages in terms of reduced complexity and improved accuracy under the assumptions of correlated signals and limited numbers of snapshots.

## 1. Introduction

The applications of wireless technology have spread into several fields, including sensor networks, environmental monitoring and public security [1,2,3]. In light of these developments, several technological policies have been created to satisfy different demands. In localization systems, the classical problem in array signal processing is finding the direction or location of sources/emitters that send signals. Thus, the incident signals on an antenna array are processed digitally to extract various types of information including their angle of arrival (AOAs) [4]. Note that the term DOA (for direction of arrival) is also used in the literature [5,6,7]. Since rescue and search services always need accurate locations of the electromagnetic beacon sources, the U.S. Federal Communications Commission (FCC) has passed a mandatory rule for location accuracy on wireless emergency calls requiring an error of no more than 125 m [8]. Additionally, accurate AOA estimation helps mobile communications and wireless positioning systems to improve their performance significantly.

A useful system that has recently appeared is smart antenna technology, which seeks efficient methods for direction finding and adaptive beam forming, integrating these algorithms with Multiple Input Multiple Output (MIMO) technology [9]. As the performance of MIMO is critically related to the propagation environment, it is vital to model the space-time path accurately in order to achieve good MIMO systems [10,11]. This technology is found especially useful in mobile communication systems through enhancing the coverage, extending the range and also increasing the capacity of systems compared to the classic antenna array set-up [12]. The AOA technique is an essential tool in controlling the space-time path, and requires accurate determination of direction of arrival at each measurement point. This aids the beamforming approach to steer the array’s beams towards wanted directions while suppressing noise and interference source signals [13]. Even small inaccuracies in estimation of angles of arrival can increase the bit error rate (BER) or cause large errors in direction when locating and tracking mobile stations, which explains the current research interest in this area [14,15].

During the past few decades, several AOA estimation methods have been used in smart antenna systems for mobile, emergency tracking position applications. The Capon or minimum variance distortion-less response (MVDR) method [16] finds the DOAs signals by estimating the power of arriving signals from one direction and considering all other signals as interference. The performance estimation of the Capon algorithm declines sharply if the source signals are correlated or when the number of snapshots is less than the number of sensors. Subspace methods, based on decomposition of the CM or observation data into signal and noise subspaces, give better estimation resolution compared with previous types. Examples of these algorithms are Multiple Signal Classification (MUSIC), the Minimum Norm method and Estimation of Signal Parameters via the Rotational Invariance Technique (ESPRIT). All these algorithms require a singular value decomposition (SVD) of the received data matrix, or an eigenvalue decomposition (EVD) of the correlation matrix. This type of algorithm provides better resolution and performance compared with the examples discussed above. MUSIC is a well-known method due to its high resolution advantage [17], however, it makes high computational demands. A Minimum Norm algorithm was proposed by Reddi [18] and then developed by Kumaresan and Tufts [19]. This method finds an optimum array weights vector that minimizes the norm of the array output, making the assumption that the first element in the weights vector is unity and other elements are zero. This algorithm has lower complexity than MUSIC but it is usable only with uniform linear arrays. The ESPRIT method supposes that the sources are narrowband signals, and also that the number of received signals is less than the number of sensors [20]: the method exploits the rotational invariance of the signal subspace, which is produced by two arrays with a translational invariance structure. It is essential to separate these subarrays translationally and not rotationally. The performances of the algorithms mentioned above deteriorate significantly when the data recorded are few or when the received signals are highly correlated. Resorting to a larger number of snapshots or removing the correlation between the incoming signals increases both complexity and execution time. Additionally, the large numbers of snapshots required are not always available in wireless environments that change rapidly.

Recent research on the DOA estimation problem has focused on Bayesian Compressed Sensing (BCS) and Sparse Signal Reconstruction (SSR) theories [21,22,23], exploring whether, if specific conditions are satisfied, it is possible to recover signals from fewer snapshots than with classical techniques [24,25]. Since the incident signals on a sensor array are intrinsically sparse in the spatial domain, the exploitation of Compressive Sensing (CS) has been extended to include the DOA estimation problem in array signal processing, where application of these methods improved estimation accuracy with fewer measurements and robustness to noise as presented in CS-MUSIC [26] and subspace-augmented (SA-) MUSIC [27]. Although these approaches offer good robustness to noise and correlated signals, their estimation performance is degraded unless there is a prior knowledge of the number of arrival signals [28]. 

The original work [24] exploited spatial sparsity while providing a method different in principle to the $\ell_{1}$-SVD approach for DOA estimation. Merging the SVD approach with the $\ell_{1}$ norm minimization based method can provide high accuracy for both narrowband and wideband scenarios. With single snapshot or single measurement vectors (SMV), the $\ell_{1}$ optimisation approach is preferred to sparse recovery because of guaranteed recovery resolution [25]. In the case of multiple snapshots, sparse signals at all measurements provide the same support. Thus, such joint sparsity is a favourable approach to improving the average recovery success under a similar uncorrelated matrix condition [29]. A compressive beamforming algorithm using CS techniques was proposed in [30] to solve the problem of DOA estimation, with a Nyquist sampling rate at the sensors. However, this is sensitive to signal correlation. Signal reconstruction from compressive measurements using an efficient technique based on CS theory called Sparse Bayesian Learning (SBL) has been proposed recently for sparse signal recovery [22]. With this SBL method, the signal recovery is formulated from a Bayesian perspective whereas the sparsity information is utilized by considering previously known sparse distributions of the incident signal at all snapshots. Note that the $\ell_{1}$-norm regularised optimisation is considered a special case of the SBL algorithm when prior Laplace signal of the impinging signals is adopted with a maximum a posteriori (MAP) optimal estimate approach. Analytical and experimental results both verify that the SBL algorithm can provide better performance than $\ell_{1}$-norm regularised optimisation [31]. In general, the measurement matrix in SBL is assumed to be accurately known; unfortunately, this assumption is invalid when perturbations on the measurement matrix are considered [32,33] or when the array manifold suffers from imperfections [34]. 

Although the CS-based techniques mentioned above give improved estimation resolution when there is a limited number of snapshots, there are still difficulties in applying them in practical situations where the correct AOAs are not located on the sampling grid. To avoid this issue and improve the estimation resolution, an extensive sampling grid is required to minimize the gap between the correct AOAs and its nearest grid point since an estimated AOA is constrained to lie on the grid. However, an extensive sampling grid results in a highly correlated matrix that breaks the required conditions for the sparse signal recovery. To overcome such issues, an off-grid sparse Bayesian inference (OGSBI) estimation algorithm is invoked [35], combining the BCS approach and the quantization error problem for point sources—the quantization error is taken to be uniformly distributed and real valued. The OGSBI method offers good estimation performance at single or limited number of measured data; however, its performance is severely degraded with low signal to noise ratio as well as when source signals are highly correlated, and with a high burden of computation where a large number of iterations are required to find the solution in some scenarios [36]. In [37], the authors propose a BCS framework approach for DOA estimation problem. This approach directly utilizes the output voltages at the sensor array to determine DOAs without need to compute the observation/sampling matrix. Two methodologies addressed in their work are single task (ST) compressive sampling and multi-task (MT) compressive sampling. These methodologies have been investigated based on BCS by using single and multiple measurements. It was found that MT-BCS gives better performance than ST-BCS in both single and multiple snapshots cases. A root-SBL method for DOA estimation problem has been proposed recently to reduce the computation complexity of SBL in [38]. In the root-SBL algorithm, the authors adopt a coarse grid and consider the sampled positions in the coarse grid as adaptable parameters. An expectation–maximization (EM) method was utilized to smooth this coarse grid iteratively and clarify that every updated grid point is achieved by the root of a certain polynomial. 

Many applications call for a high resolution and low complexity AOA estimation method in these conditions. The contribution of this present paper is to propose a simple and low complexity method that can be used to estimate the DOAs of signals efficiently; the proposed method is called the propagator direct data acquisition (PDDA) method. The PDDA algorithm is based on computing the propagator vector which represents the cross correlation between the received data from the first sensor and the other sensors. This propagator vector retains all the information about how the phasors of signals arriving from various directions sum at each sensor, which has the effect of normalizing to the phase of the first sensor and eliminating the dependency on the signal time series, which in turn improves robustness to noise. Consequently, the DOA signals can be estimated efficiently with a single snapshot or a few snapshots even when the impinging sources are highly correlated. Furthermore, it does not require prior knowledge of the number of arriving signals, unlike MUSIC, ESPRIT and other techniques. The new method estimates the directions of the received signals directly from the measured data without the need to construct the covariance matrix, to invert the matrix or to use EVD/SVD approaches. This decreases the complexity considerably. Computational complexity is investigated in terms of the required number of computational operations and the execution time, the comparison results demonstrating that the PDDA method is efficient computationally. The proposed method is implemented by using linear and circular sensor arrays to perform 1D and 2D estimation; many numerical examples are presented to show its performance. It is compared with several popular and recent AOA methods in terms of numbers of snapshots collected, behaviour with SNR, correlation of signals sources and execution time. An intensive Monto Carlo simulation is achieved with random angles over different scenarios, with the identical conditions applied to all algorithms to ensure a fair comparison. The experimental results show that the proposed algorithm performs best in the many scenarios and is comparable in other conditions to the best alternative algorithms, moreover with lower complexity. 

The rest of the paper is organized as follows: Section 2 reviews AOA modelling in both linear and circular arrays. The mathematical model of the proposed method and the computational complexity between PDDA and other techniques are presented in Section 3. Simulation results, discussion and comparisons between PDDA and other AOA methods appear in Section 4. Finally, Section 5 summarizes the results and sets out conclusions. 

The following notation is used in the remainder of this paper: lower-case and upper-case bold characters denote vectors and matrices, respectively, whereas the lower-case characters refers to scalar values. E {·} represents the statistical expectation. (·)^T^ and (·)^H^ indicates to the transposition and conjugate transposition of a vector or a matrix. $\left(\hat{.}\right)$ represents the estimated value.

## 2. Array Signal Model

**Figure 1:**
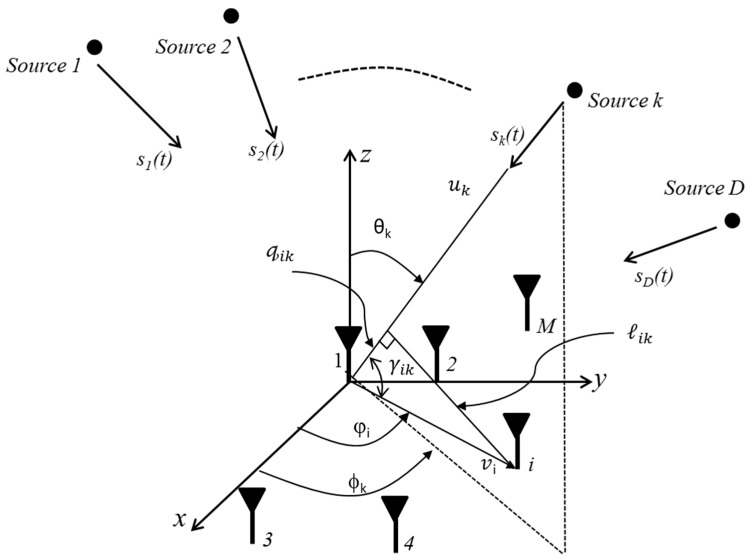
M-Arbitrary element array with D arriving signals.

The AOA estimation setup can be explained with reference to Figure 1. Suppose D is the number of incoming signals, S(t), arriving from different directions and received by M sensors. The time sample of each component, X(t), of the received signal contains AWGN, N(t), and can be defined by:

(1) X(t) =A(θ) S(t)+N(t)

where $A\left(\theta \right)=\left[\;a(\theta_{1\;})\;a(\theta_{2\;})\ldots \;a(\theta_{D\;})\right]\;$ is the arriving signal steering matrix for *D* signals, *k* = 1, 2, …, *D*. $S\left(t\right)={\left[s_{1}\left(t\right),\;s_{2}\left(t\right),\;\ldots ,\;s_{D}\left(t\right)\right]}^{T}$ is the (*D* × *N*) incident signals, $\;N\left(t\right)=\left[n_{1}\left(t\right),\;n_{2}\left(t\right),\;\ldots ,\;n_{M}\left(t\right)\right]\;$ is an array of AWGN for each channel, $a\left(\theta_{k}\right)=\left[\begin{array}{ccc} 1 & e^{j\beta dsin\theta_{k}} & \begin{array}{ccc} \cdots & \cdots & e^{j\beta d\left(M-1\right)sin\theta_{k}} \end{array} \end{array}\right]$ is the steering vector of a linear array, d is the separation between adjacent elements and $\beta =\frac{2\pi }{\lambda }$ is the spatial frequency.

A circular array is a common geometry which can be used to increase the gain. Also, it provides beam steering in three dimensions that is not possible with a linear array. A uniform circular array of M elements in the x-y plane is assumed in this work. With the circular array, both elevation angle (θ) and azimuth angle (ϕ) can be estimated: the total received signal is:

(2) $$X\left(t\right)=A(\theta ,{\;}_{\;}\phi )\;S\left(t\right)+N\left(t\right)$$

where $A(\theta ,{\;}_{\;}\phi )$ is the *M* × *D* matrix of steering vectors:

(3) $$A(\theta ,{\;}_{\;}\phi )=\;\left[\;a(\theta_{1},{\;}_{\;}\phi_{1})\;\;\;\;a(\theta_{2},{\;}_{\;}\phi_{2})\;\;\;\ldots \;\ldots \;\;\;\;a(\theta_{D},{\;}_{\;}\phi_{D})\;\right]$$

Consider there are *k* sources incident on the sensor array as shown in Figure 1, then the unit vectors that include the direction of $\theta_{k}$ and $\phi_{k}$ can be defined as follows:

(4) $$u_{k}=\cos\phi_{k}\sin\theta_{k}{\hat{a}}_{x}+\sin\phi_{k}\sin\theta_{k}{\hat{a}}_{y}+\cos\theta_{k}{\hat{a}}_{z}$$

where ${\hat{a}}_{x},\;{\hat{a}}_{y}\;$ and ${\hat{a}}_{z}$ are the unit vectors for Cartesian co-ordinates. With a ring array located on the x-y plane, where *r* is the array radius and phase angle ($\varphi_{i}$), the unit vector from the original reference point to the *i*th element is given as follows:

(5) $$v_{i}=r_{i}\cos\varphi_{i}{\hat{a}}_{x}+r_{i}\sin\varphi_{i}{\hat{a}}_{y},\;i=\;1,\;2,\;\ldots ,\;M.$$

(6) $$\varphi_{i}=\frac{2\pi }{M}\left(i-1\right)$$

The angle ($\gamma_{ik}$) can be obtained from the dot product between unit vectors $v_{i}$ and $u_{k}$ as shown below:

$$\gamma_{ik}=\cos^{-1}\left(\frac{v_{i}.u_{k}\;}{∥v_{i}∥.∥u_{k}∥}\right)$$

$$\gamma_{ik}=\cos^{-1}\left(\frac{\left(\cos\varphi_{i}{\hat{a}}_{x}+\sin\varphi_{i}{\hat{a}}_{y}\right)\cdot \left(\cos\phi_{k}\sin\theta_{k}{\hat{a}}_{x}+\sin\phi_{k}\sin\theta_{k}{\hat{a}}_{y}\right)}{∥v_{i}∥.∥u_{k}∥}\right)$$

$$\gamma_{ik}=\cos^{-1}\left(\frac{\cos\varphi_{i}\cos\phi_{k}\sin\theta_{k}+\sin\varphi_{i}\sin\phi_{k}\sin\theta_{k}}{∥v_{i}∥.∥u_{k}∥}\right)$$

$$\gamma_{ik}=\cos^{-1}\left(\frac{\sin\theta_{k}\cos(\phi_{k}-\varphi_{i})}{∥v_{i}∥.∥u_{k}∥}\right)$$

(7) $$\gamma_{ik}=\cos^{-1}\left(\sin\theta_{k}\cos(\phi_{k}-\varphi_{i})\right)$$

φ is vector with dimension (1 × *M*) as given below:

(8) $$\varphi =\left[\varphi_{1}\;\varphi_{2},\ldots \ldots ,\;\;\varphi {\;}_{M}\right]$$

while γ is the matrix with dimension (M×D) given as follows:

(9) $$\gamma =\;\left[\begin{array}{c} \begin{array}{c} \gamma_{11}\\ \gamma_{21} \end{array}\\ \vdots \\ \gamma_{M1} \end{array}\;\;\;\;\;\;\;\begin{array}{c} \begin{array}{c} \gamma_{12}\\ \gamma_{22} \end{array}\\ \vdots \\ \gamma_{M2} \end{array}\;\;\;\;\;\;\;\begin{array}{c} \begin{array}{c} \cdots \\ \vdots \end{array}\\ \ddots \\ \ldots \end{array}\;\;\;\;\;\;\;\;\begin{array}{c} \begin{array}{c} \gamma_{1D}\\ \gamma_{2D} \end{array}\\ \vdots \\ \gamma_{MD} \end{array}\;\right]$$

In order to compute the phase difference, the time difference of arrival of the *k*th signal at each reference element and each sample element needs to be calculated. The wavefront time delay is calculated using the difference in distance (${𝓆}_{ik}$) that is given by:

(10) $${𝓆}_{ik}=r\cos\gamma_{ik}=\;r\cos\left(\cos^{-1}\left(\sin\theta_{k}\cos(\phi_{k}-\varphi_{i})\right)\right)=r\sin\theta_{k}\cos(\phi_{k}-\varphi_{i})$$

where r can be computed as follows:

(11) $$r=\frac{d}{2\sin\left(\frac{2\pi }{M}\right)}$$

The phase difference ($\psi_{ik}$) can be expressed in the following formula:

(12) $$\psi_{ik}=\beta .{𝓆}_{ik}=\frac{2\pi }{\lambda }r\sin\theta_{k}\cos(\phi_{k}-\varphi_{i})$$

Then, the circular array steering vector can be defined as follows:

(13) $$a(\theta_{k},{\;}_{\;}\phi_{k})=\left[\begin{array}{c} e^{-j\psi_{1k}}\\ e^{-j\psi_{2k}}\\ \begin{array}{c} \vdots \\ \vdots \\ e^{-j\psi_{Mk}} \end{array} \end{array}\right]$$

As arriving signals are time-varying, the calculations are based on time samples of the arriving signal. Clearly, if the signal sources are moving from one location to another, the corresponding received angles and the matrix of steering vectors change with time. The array covariance matrix ($R_{xx}$) can be expressed in the following form:

$$R_{xx\;}=E\left[X\left(t\right)\;X^{H}\left(t\right)\;\right]=E\left[AS\left(t\right)S^{H}\left(t\right)A^{H}\right]+E\left[N\left(t\right)\;N^{H}\left(t\right)\right]$$

(14) $$R_{xx\;}=AR_{ss\;}A^{H}+\sigma_{n}{}^{2}I_{M}$$

where $R_{ss\;}$ is the (*D* × *D*) source signal correlation matrix $R_{ss}=E\left[S\left(t\right)S\left(t\right)\right]$, $\sigma_{n}{}^{2}$ is the noise variance and $I_{M}$ is the *M* × *M* identity matrix. In this case, the received signals are taken as being uncorrelated, so that $R_{ss\;}$ is a diagonal matrix. However, if the received signals are correlated, the signal correlation matrix will be non-diagonal, as appears in realistic environments due to multipath propagation or unfriendly jamming signals. Complete knowledge of $R_{xx\;}$ cannot be assumed; instead, we may use as necessary the sample-average estimated array input autocovariance matrix given by:

(15) $${\hat{R}}_{xx}\approx \frac{1}{N}\overset{N}{\underset{k=1}{\sum }}X\left(t\right)X^{H}\left(t\right)\;$$

After the CM is obtained, AOA estimation is performed using a suitable AOA algorithm.

## 3. Proposed Algorithm

The received data matrix can be used to estimate the AOA instead of using the CM. The proposed algorithm depends on computing the propagator vector p, which represents the cross correlation between the first row (received signal data from the first element) and the other rows. This has the effect of normalizing to the phase of the first element and removing the dependency on the signal time series. The propagator vector therefore retains all the information about how the phasors of signals arriving from various directions sum at each sensor. Correlation with the steering vector thus elicits the individual AOAs. It also reduces the computational complexity compared to calculating the entire CM. 

Suppose that the data matrix X(t) contains *N* samples of data obtained from *M* sensors. X(t)=AS(t)+N(t) is an *M* × *N* matrix (***X***_*m,n*_ $\in C^{1\times 1}\;)\;$ and can be written as follows:

(16) $$X\left(t\right)=\;\left[\begin{array}{c} \begin{array}{c} x_{1}\left(t_{1}\right)\\ x_{2}\left(t_{1}\right)\\ \vdots \end{array}\\ \vdots \\ x_{M}\left(t_{1}\right) \end{array}\;\begin{array}{c} \begin{array}{c} x_{1}\left(t_{2}\right)\\ x_{2}\left(t_{2}\right)\\ \vdots \end{array}\\ \vdots \\ x_{M}\left(t_{2}\right) \end{array}\;\begin{array}{c} \begin{array}{c} \cdots \\ \vdots \\ \ddots \end{array}\\ \vdots \\ \ldots \end{array}\;\begin{array}{c} \begin{array}{c} \cdots \\ \vdots \\ \vdots \end{array}\\ \ddots \\ \ldots \end{array}\;\begin{array}{c} \begin{array}{c} x_{1}\left(t_{N}\right)\\ x_{2}\left(t_{N}\right)\\ \vdots \end{array}\\ \vdots \\ x_{M}\left(t_{N}\right) \end{array}\right]$$

**Figure 2:**
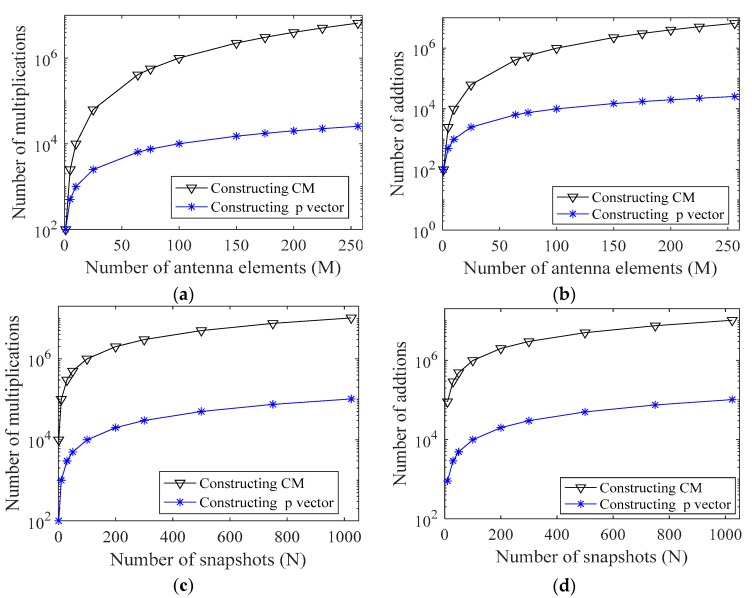
Comparison of complexity for covariance matrix (CM) and propagator vector (p) construction. (**a**) Multiplications required for *N* = 100 and *M* = 1:256; (**b**) Additions required for *N* = 100 and *M* = 1:256; (**c**) Multiplications required for *M* = 100 and *N* = 1:1000; (**d**) Additions required for *M* = 100 and *N* = 1:1000.

In order to compute the propagator vector p we divide the *X* matrix into two sub-matrices as follows:

(17) $$h=\;\left[\begin{array}{cc} x_{1}\left(t_{1}\right) & x_{1}\left(t_{2}\right) \end{array}\;\cdots \;\cdots \;x_{1}\left(t_{N}\right)\right]$$

(18) $$ℋ=\left[\begin{array}{c} \begin{array}{c} x_{2}\left(t_{1}\right)\\ \vdots \end{array}\\ \vdots \\ x_{M}\left(t_{1}\right) \end{array}\;\begin{array}{c} \begin{array}{c} x_{2}\left(t_{2}\right)\\ \vdots \end{array}\\ \vdots \\ x_{M}\left(t_{2}\right) \end{array}\;\begin{array}{c} \begin{array}{c} \cdots \\ \ddots \end{array}\\ \vdots \\ \ldots \end{array}\;\begin{array}{c} \begin{array}{c} \cdots \\ \vdots \end{array}\\ \ddots \\ \ldots \end{array}\;\begin{array}{c} \begin{array}{c} x_{2}\left(t_{N}\right)\\ \vdots \end{array}\\ \vdots \\ x_{M}\left(t_{N}\right) \end{array}\;\right]$$

where h represents the first row of the matrix X whereas ℋ represents the reset of X. Now, compute vector p, as follows:

(19) $$p=h{ℋ}^{H}/hh^{H}$$

p  gives the cross-correlation of the time-series from each sensor element with that from the first element, with size 1 × (*M* − 1). We now add a unit element representing the correlation of the first row with itself. This yields:

(20) $$℮={\left[\begin{array}{cc} 1 & p \end{array}\right]}^{T}$$

where  ℮ is a vector of size 1 × *M*. The complexity of localization systems depends on hardware, software and operating factors. The PDDA technique presents lower computational complexity, as can be verified by comparing the number of computational operations required to construct the CM, which is used in most AOA methods, and that required to compute the vector (p) utilized in the PDDA method, as shown in Table 1 and Figure 2.

After computing CM, some methods need to calculate the inverse of the CM whereas other need to decompose it, with prior knowledge of the number of arrival signals. Table 2 gives a comparison between PDDA and some common AOA methods based on the above-mentioned criteria. 

Then, the spatial power spectrum can be obtained from vector (℮) as follows:

(21) $$P\left(\theta \right)={\left|a\left(\theta \right)\;℮\right|}^{2}$$

The complexity of the required number of multiplications and additions in the scanning angle process stage to construct the spatial spectrum differs from one method to the other. Table 3 compares the complexity of the PDDA and other popular AOA methods at this stage as described below.

To obtain narrower peaks and also to minimize side-lobe levels, we propose the following approach: first find the maximum point in P(θ):

(22) 𝓌=max(P(θ))

where 𝓌 is a scalar value. The second step is subtracting the value of the global maximum from the other points in Equation (21). This yields the following equation:

(23) $$P_{s}\left(\theta \right)=𝓌-P\;\left(\theta \right)$$

Once this condition is achieved, we will obtain nulls in the direction of incoming signals. The PDDA method can be expressed in a new formula to acquire maximum peaks in the AOA as follows:

(24) $$P_{PDDA}\;\left(\theta \right)=\frac{1}{P_{s}\left(\theta \right)+\varepsilon }$$

where ε is a small scalar value added in order to avoid possible singularities. Equation (24) is highly non-linear, so it exaggerates signals close to 𝓌 and suppresses side-lobes. With an appropriate choice of ε, it is then straightforward to apply a threshold value to separate genuine peaks from side-lobes. The simulation steps of this method are illustrated as follows:

**Algorithm 1:** Propagator Direct Data Acquisition (PDDA) for 1D and 2D Direction Estimation**Input:** the received signals $X\in C^{M\times N}$, with *M* sensors, *N* number of snapshots, *D* source signals. **Output:** The estimated AOAs. Step 1: Compute the received signal and add noise with a certain SNR (X(t) =AS(t)+N(t)) Step 2: Divide the received signal matrix into two portions as given in Equations (17) and (18) Step 3: Construct the propagator vector (p) by applying Equation (19). Step 4: Compute the vector (℮) by using Equation (20). Step 5: Construct the pseudospectrum of the proposed method by scanning ℮ through the whole scanning range of the *θ* plane with a specific scanning Step , δ, thus:  **for** ii = 0:δ:*θ* $P_{PDDA}\left(\mathrm{ii}\right)={\left|\;a\left(\theta \right)\;℮\right|}^{2}$ **end** If this method is applied for a circular array, the vector ℮ needs to be scanned over both the *θ* and ϕ planes with δ as follows: **for** ii = 0:δ:*θ* **for** jj = 0:δ:ϕ $P_{PDDA}\left(\mathrm{ii},\mathrm{jj}\right)={\left|a\left(\theta ,\phi \right){\;}^{H}\;℮\right|}^{2}$ **end** **end** Step 6: Find the maximum global point in the spatial spectrum (i.e., 𝓌). Step 7: Subtract the maximum value from the other values by applying Equation (23). Step 8: Plot the pseudo-spectrum of the PDDA by using Equation (24) and set  ε=0.01. Step 9: Find the locations of the peaks to detect the arrival angles.

The overall computational cost of the mentioned above algorithms are calculated and described below in Table 4. It is obvious from Table 1, Table 2, Table 3 and Table 4 that the PDDA method has lower computation complexity than the other AOA methods presented.

## 4. Numerical Simulations and Discussion

To demonstrate and verify the theoretical claims of the proposed method, simulations with both uniform linear arrays and uniform circular arrays are carried out.

### 4.1. Uniform Linear Array (ULA)

**Figure 3:**
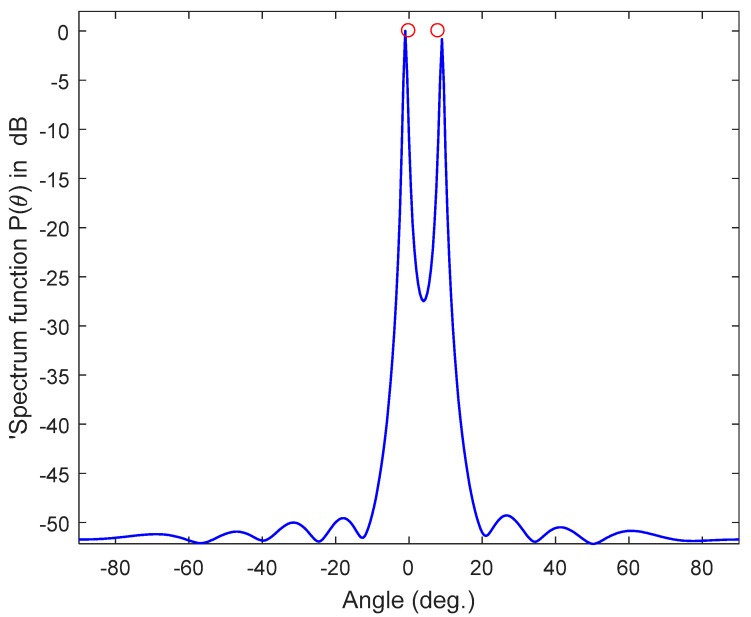
Performance estimation of the PDDA method, *M* = 10, SNR = 10 dB, *N* = 10, *D* = 2.

This simulation parameters are SNR = 10 dB, number of samples *N* is 10, and the inter-element spacing is *d* = 0.5λ. The scanning step angle is 0.5°. Figure 3 shows an example of two signals incident on a ULA with *M* = 10 sensors, arriving from different directions (θ = 0° and 8°). As can be seen from Figure 3, the PDDA method estimates the angles of the arrival signal accurately, and also provides sharp peaks in the directions of arrival, with negligible side lobes.

### 4.2. Uniform Circular Array (UCA)

Some applications such as radar and tracking systems require estimation of both elevation and azimuth angles in order accurately to track targets that are moving in three dimensions. Hence, the proposed method is next applied to the circular array, with a range of azimuth angles [0, 360°] and elevation angles [0, 90°]. The type of received signals is BPSK with carrier frequency f_c_ = 10 GHz and the signals are distorted with AWGN. The other simulation parameters are the number of sensors *M* = 15, the number of samples is 100, SNR = 10 dB, and *d* = 0.5λ. Three cases are considered in this simulation, with the directions of arrival generated randomly. 

**Figure 4:**
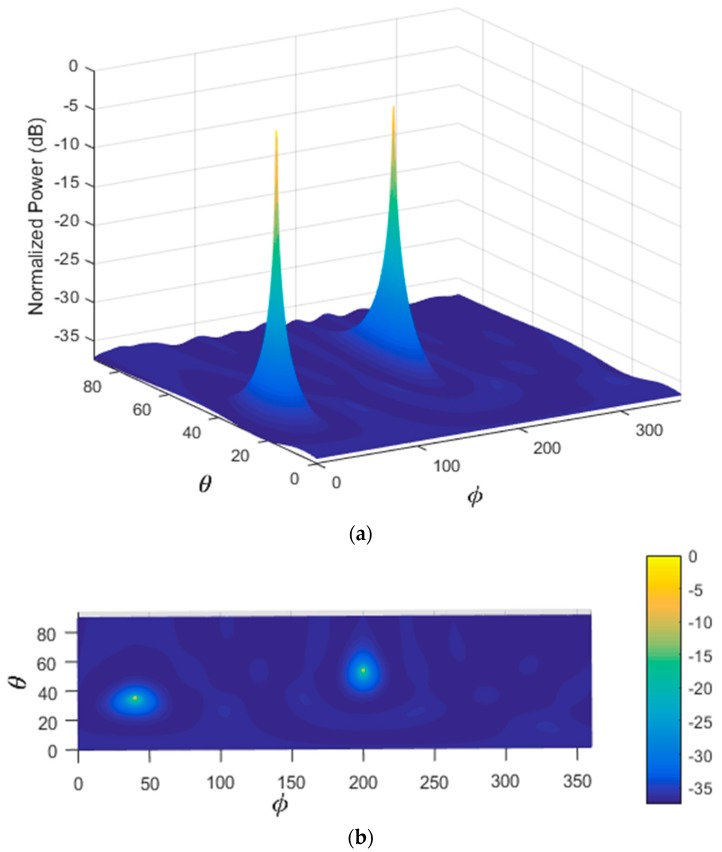
Performance estimation of the PDDA method with two AOAs. (**a**) 3D plot of the PDDA method; (**b**) 2D plot of the PDDA method.

**Figure 5:**
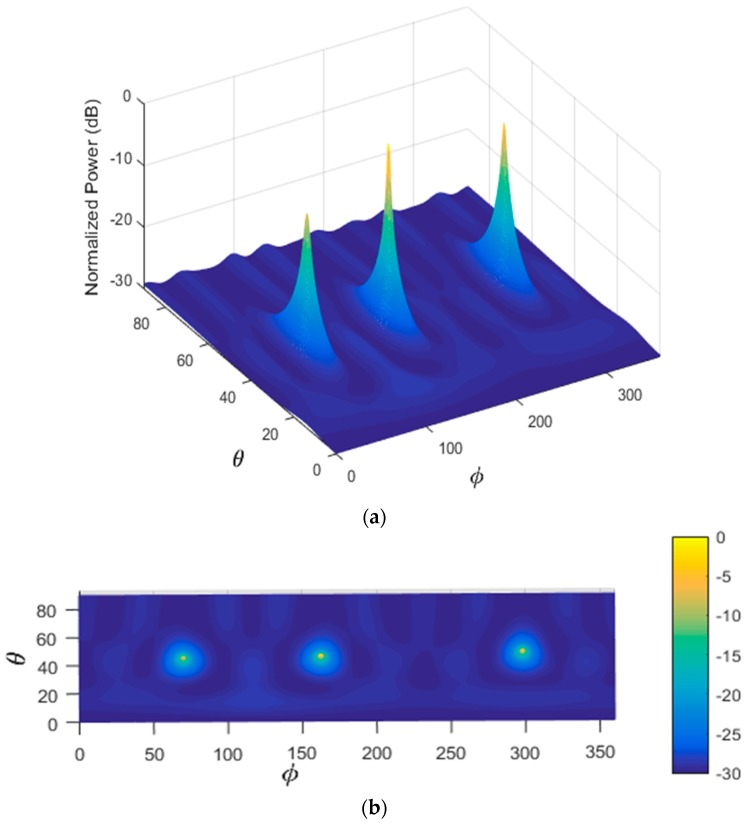
Performance estimation of the PDDA method with three AOAs. (**a**) 3D plot of the PDDA method; (**b**) 2D plot of the PDDA method.

**Figure 6:**
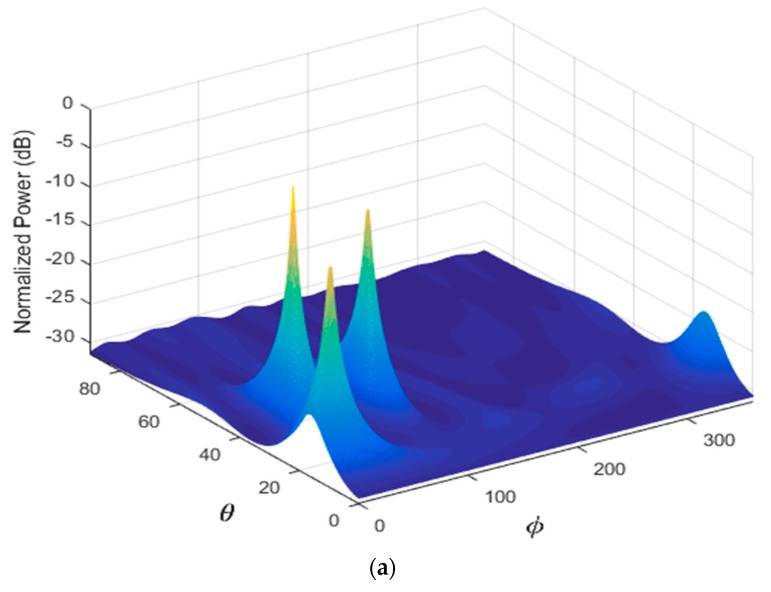

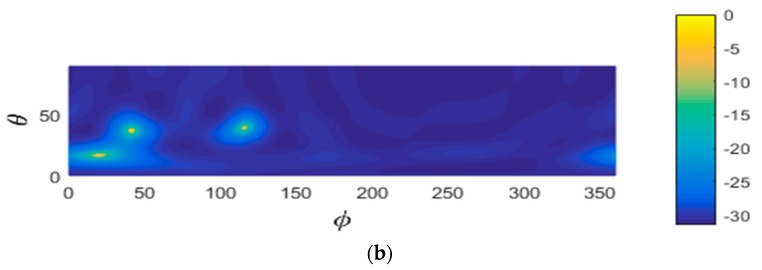
Performance estimation of PDDA method with three AOAs close together. (**a**) 3D plot of the PDDA method; (**b**) 2D plot of the PDDA method.

In the first scenario, two signals incident on the sensor array from different directions are considered as shown in Table 5. Figure 4 illustrates the performance estimation of the PDDA method for case 1. In the second scenario, there are three signals arriving from different directions as presented in Table 5, case 2. The performance of the PDDA method and its precision in this case are illustrated in Figure 5. The last simulation example considers signals clustered closely together as given in Table 5, case 3, with results shown in Figure 6. It is clear from these graphs that the proposed method gives accurate and sharp peaks in the directions of the targets.

### 4.3. Comparison with Other AOA Methods

In this section, the performance of the PDDA method is compared with four commonly used AOA methods namely: MVDR, MUSIC, Min Norm, ESPRIT; and also with two algorithms which have been proposed recently, namely OGBSI and Root-SBL. The simulation codes of some methods are provided by MATLAB R2016b, for example the MUSIC algorithm and the Capon algorithm [43]. Four scenarios are considered in this comparison namely: number of snapshots taken, signal to noise ratio (SNR), the correlation between sources of the incident signals and the execution time. For the first three scenarios, the average root mean square error (ARMSE) is computed for each criterion. The ARMSE can be defined as follows:

(25) $$\mathrm{ARMSE}=\frac{1}{K}\sum_{j=1}^{K}\sqrt{\frac{1}{D}\sum_{k=1}^{D}\left[{\left(\theta_{k}-{\hat{\theta }}_{k}\right)}^{2}\right]\;}$$

where *D* is the number of signals arriving, $\theta_{k}$ is the actual angle, ${\hat{\theta }}_{k}$ is the estimated angle and *K* is the number of Monto Carlo simulation trials. For simplicity in calculating each RMSE, we proceed as follows. We have *D* actual DOAs and L estimated DOAs. In the practical applications or even in the simulation scenarios, the number of detected peaks can be fewer or more than actual DOAs. Three scenarios are possible namely: *D* = L, *D* < L, *D* > L. In the case of L > *D* (because of false peaks), we include only the estimates for the *D* loudest peaks. Then, in all cases, we calculate the L × *D* array of angle error magnitudes between each pair of actual and estimated DOAs and then take the minimum for each of *D* columns and average the root mean square values.

#### 4.3.1. Number of Snapshots

**Figure 7:**
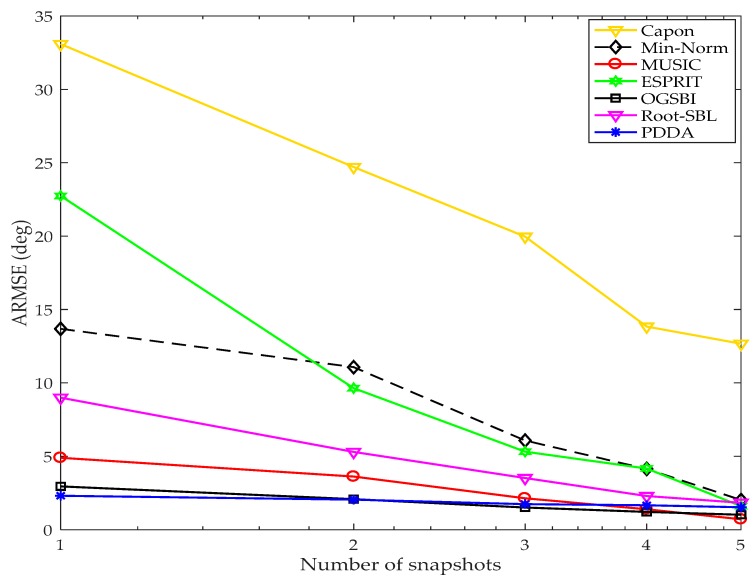
Average root mean square error (ARMSE) vs. number of snapshots with signal to noise ratio (SNR) = 10 dB, *M* = 8, *D* = 2, 1000 Monto Carlo simulations for each *N*.

Typically, resorting to a larger number of snapshots is not always practical in wireless environments since the sources of signals are changing rapidly. This simulation compares the estimation accuracy of the proposed method with other AOA methods for obtaining a reliable estimation of DOAs signals using a limited number of snapshots. A ULA with *M* = 8 elements with half wave spacing between elements is selected. Two BPSK signals (*D* = 2) are incident on this array from different directions—these signals are corrupted with AWGN and the SNR is 10 dB. Five different numbers of snapshots of the received signals are collected in this experiment namely: *N* = 1, 2, 3, 4 and 5. A thousand trials of two AOAs are randomly generated, each set being applied to all AOA methods. The ARMSE of every *N* is calculated and plotted for all techniques as shown in Figure 7. Clearly, the PDDA method gives the best resolution compared with other AOA methods for a single snapshot. With *N* = 2 and 3, the performance estimation of PDDA and OGBSI are convergent and they are the best compared with the other AOA methods. At *N* = 4, PDDA, OGBSI and MUSIC have roughly the same estimation accuracy and are better than the other algorithms. At *N* = 5, the estimation resolution of each method can be sorted in descending order as follows: MUSIC, OGSBI, PDDA, ESPRIT, Root-SBL, Min-Norm and Capon. So, the performance of the proposed method outperforms all methods with a single snapshot and is comparable to MUSIC and OGSBI for other numbers of snapshots. The reason for poor accuracy for the Capon method is that when *N* is less than *M*, the Capon method suffers from singularities due to matrix inversion operations.

#### 4.3.2. Array-Signal to-Noise (SNR)

**Figure 8:**
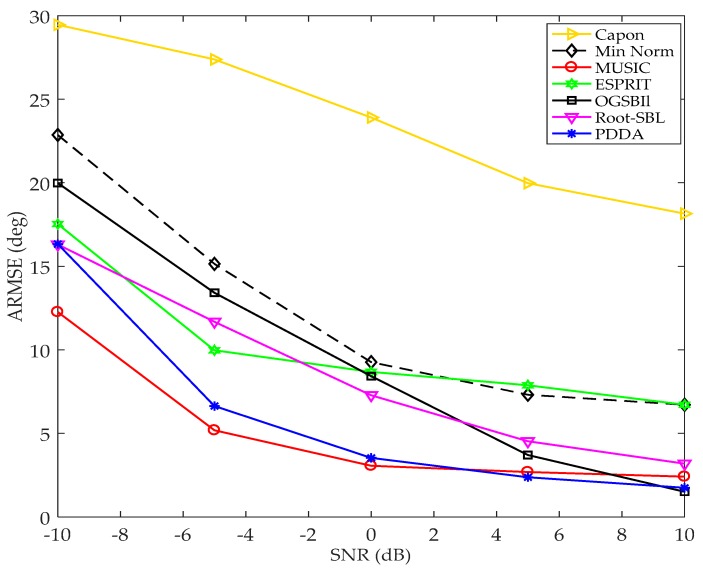
ARMSE vs. SNR, with *M* = 8, *D* = 2, *N* = 3, 1000 Monto Carlo simulations for each SNR.

In this scenario, we still assume the number of snapshots measured is limited (*N* = 3) but with differing SNR. The SNR at the input to the sensor array receiver plays a crucial role in the performance of AOA in localization systems. Therefore, this simulation compared the variations in performance of AOA methods with a variation of SNR. A ULA consisting of *M* = 8 sensors and *d* = 0.5λ is considered, this array receiving two BPSK signals from different directions. 1000 Monto Carlo simulations with *D* = 2 are used to generate AOAs: these angles are applied for all algorithms. The ARMSE is computed for each SNR and plotted for all techniques in Figure 8. The effect of the SNR on the performance estimation of each technique can be clearly seen in this graph: at low SNR (i.e., SNR less than 0 dB), MUSIC and PDDA methods give the best accuracy compared with other AOA techniques. However, as the SNR improves the PDDA method gives the best estimation resolution compared with all the AOA techniques presented. The reason that MUSIC has better accuracy than the PDDA method at poor SNR is because the large amount of noise diffuses the phasors of arriving signals and this in turn affects negatively normalizing the phase of the first sensor and eliminating the dependency on the signal time series. On the other hand, MUSIC has higher complexity than PDDA as shown in Table 4.

#### 4.3.3. Correlation

**Figure 9:**
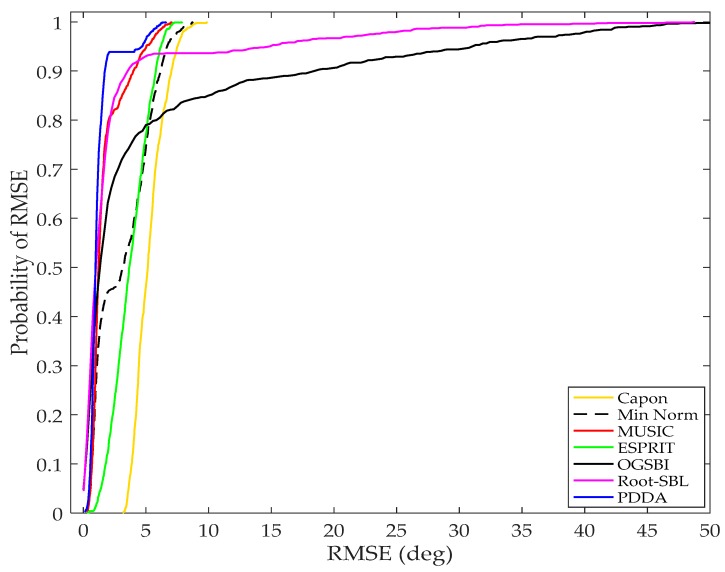
Error probability vs RMSE, *M* = 8, *D* = 2, *N* = 10, SNR = 1 dB, CC = 0.95.

Correlation or similarity between the arriving signals impacts negatively on the performance of direction estimation systems. A simulation is next run for two correlated received signals with correlation coefficient (CC = 0.95) incident on a linear array with *M* = 8 elements and *d* = 0.5λ. The other simulation parameters are number of samples, *N* = 10, and SNR = 1 dB. In order to ensure a fair and comprehensive comparison, a thousand sets with pairs of AOAs are randomly generated, each set being applied to all algorithms. The RMSE is computed for each trial and then plotted as a cumulative distribution function (CDF). As can be seen from Figure 9, the PDDA presents the best accuracy among the AOA methods and this demonstrates that the proposed method has good robustness and less sensitivity to correlation of source signals. It has been also observed that OGSBI and Root-SBL, based on Bayesian compressed sensing and sparse signal reconstruction principles, have high sensitivity to correlated signals. MUSIC still gives a good performance, whereas Min Norm, ESPRIT and MVDR algorithms perform poorly under such correlation conditions.

#### 4.3.4. Execution Time

**Figure 10:**
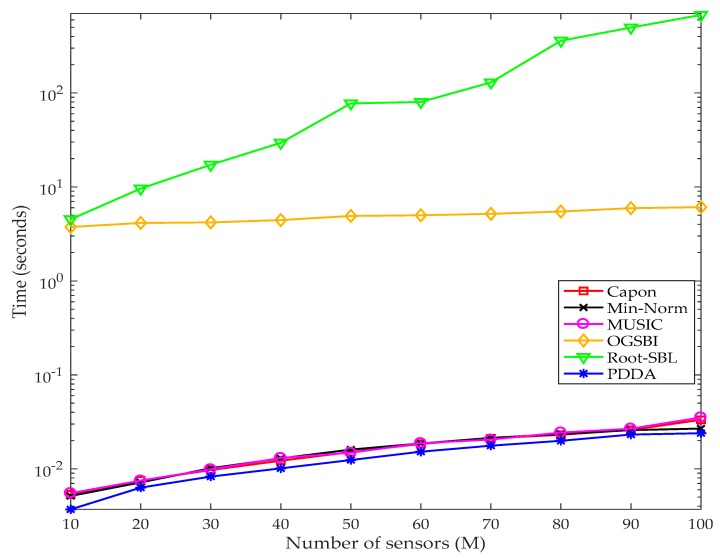
Execution time comparison based on different numbers of sensors.

The speed of computation is a crucial factor for any application, hence the time for execution of the PDDA method is compared with state-of-the-art AOA methods in identical conditions. Since the computation complexity shown in table 4 mainly depends on the number of the sensors (M), the comparison of the execution time has been implemented by changing M = [10, 20, …, 100] and keeping the other simulation parameters constant. A MATLAB simulation was run with one hundred iterations (i.e., K = 100) for each method, and the average times of execution at each M recorded using tic and toc functions. The angular range of interest is [−90°, 90°] with interval grid δ = 0.5°, *D* = 6, SNR = 10 dB and *N* = 100. For the OGSBI and Root-SBL methods, the tolerance error and maximum number of iterations are set at 0.001 and 500 respectively, while the other simulation parameters for these are as given in [35,38] respectively. All the experiments have been carried out in MATLAB R2016-b on a PC with a Windows 8.1 operating system, processor: Intel(R) Core (TM) i7-4790 CPU @ 3.6 GHz, with 32 GB installed RAM. It is obvious from Figure 10 that the PDDA method is fastest. Minimum Norm, MUSIC and Capon give reasonable speed, whereas the Root-BSL and OGBSI techniques are the slowest. This is because their computational complexity not only depends on the number of sensors but also on the other parameters such as tolerance error and maximum number of iterations, which are required to find the optimum solution. The exact execution time of each method may differ from these results according to the specifications of the computer and situations which are associated with program running but the relative behaviour for each algorithm should be the same.

## 5. Conclusions

A new and low complexity angle of arrival method, which estimates the AOAs of the received signals directly from the received data without the need to construct the CM, is proposed in this paper. The reduction in complexity of the proposed method has been demonstrated mathematically and then explained in terms of the number of computational operations. The reduction of complexity has been achieved through avoiding constructing the CM and computing the matrix inverse or applying eigenvalue decomposition. The PDDA was implemented with two types of sensor arrays: uniform linear sensor array and circular sensor array. Many simulation examples illustrate the performance estimation of the new algorithm in comparison with well-assessed state-of-the-art AOA estimation methods over a wide range of scenarios. Results show that the new method is suitable for a single or low number of snapshots, has a lower computational cost than existing techniques, and works well with correlated signals. 

## Figures and Tables

**Table 1:** Computational operations required to construct the CM compared with the p vector construction.

Method	Number of Multiplications	Number of Additions	Divisions
PDDA based on vector (p)	*M* × *N*	*M* × (*N* − *M*)	*M* − 1
Computing CM	*M*^2^ × *N*	*M*^2^ × (*N* − 1)	None

**Table 2:** Showing the complexity comparison between the propagator direct data acquisition (PDDA) and other angle of arrival (AOA) techniques.

Method	Covariance Matrix | Inverse Required?	Eigen-Decomposition (EVD) Required?	Knowledge of the Number of Arriving Signals Required?
Capon [16]	Yes	No	No
Maximum Entropy (ME) [39]	Yes	No	No
MUSIC [17]	No	Yes, in order to decompose (*M* × *M*) matrix	Yes
Min-Norm [19]	No	Yes	Yes
Pisarenko [40]	No	Yes	No
ESPRIT [27]	No	Yes, for decomposition of an (*M* × *M*) matrix and a (*D* × *D*) matrix	Yes
Root MUSIC [41]	No	Yes	Yes
Propagator [42]	No but it needs to compute matrix inverse with size (*D* × *D*)	No	Yes
PDDA	No	No	No

**Table 3:** Showing the computational operations required to construct the spatial spectrum for the PDDA and popular AOA methods.

Method	Number of Multiplications	Number of Additions
Capon	M × (M + 1)	(M − 1) × M + (M − 1)
MUSIC	(M − D) × (M + 1)	(M − D) × (M − 1) + (M – D − 1)
Pisarenko	M	M − 1
Propagator	M × (M + 1)	(M − 1) × M + (M − 1)
PDDA	M	M − 1

**Table 4:** Showing the overall computational complexity comparison between PDDA and popular AOA methods.

Method	Computational Complexity
Capon	O(*M*^2^ *N + M*^3^ + *M*^2^(180/δ))
MUSIC	O(*M*^2^ *N + M*^3^ + *M*^2^(180/δ))
ESPRIT	O(*M*^2^ *N + M*^3^ + *D*^3^)
Min-norm	O(*M*^2^ *N + M*^3^ + *M*(180/δ))
Pisarenko	O(*M*^2^ *N + M*^3^ + *M* (180/δ))
ME	O(*M*^2^ *N + M*^3^ + *M* (180/δ))
Propagator	O(*M*^2^ *N + M*^2^ D + *M*^2^ (180/δ))
OGSBI	O(max(*M* (180/δ)^2^, *M N* (180/δ)) per iteration
PDDA	O(*M N + M* (180/δ))

**Table 5:** Directions of arrival signals.

Case	Elevation Angles (*θ*)	Azimuth Angles (*ϕ*)
1	32°	40°
	50°	200°
2	42°	69°
	44°	163°
	46°	298°
3	39°	115°
	36°	40°
	17°	20°
